# An explainable machine learning framework integrating clinical and lipidomic signatures for early severity stratification of hypertriglyceridemic pancreatitis

**DOI:** 10.3389/fmed.2026.1880402

**Published:** 2026-08-10

**Authors:** Yanrong Yao, Bo Yuan, Kai Wang, Chensi Zhao, Jiazheng Yang, Jingli Liu, Zuozheng Wang, Yujing Gao

**Affiliations:** 1 Key Laboratory of Fertility Preservation and Maintenance of Ministry of Education, Department of Biochemistry and Molecular Biology, School of Basic Medical Sciences, Ningxia Medical University, Yinchuan, China; 2 Department of Hepatobilology, General Hospital of Ningxia Medical University, Yinchuan, China; 3 The First Clinical Medical College, Ningxia Medical University, Yinchuan, China; 4 Outpatient Department, General Hospital of Ningxia Medical University, Yinchuan, China; 5 School of Public Health, Ningxia Medical University, Yinchuan, China

**Keywords:** explainable artificial intelligence, hypertriglyceridemia-induced pancreatitis, KEGG pathway analysis, lipidomics, machine learning, severity stratification, SHAP

## Abstract

**Background:**

Hypertriglyceridemic pancreatitis (HTGP) is increasingly recognized as a major etiology of acute pancreatitis and is associated with a higher risk of early severe progression, systemic complications, and mortality compared with other etiologies. However, currently available scoring systems demonstrate limited accuracy and poor mechanistic interpretability in HTGP.

**Aim:**

To develop and validate an explainable machine learning model for early stratification of severe progression in HTGP and to explore its underlying lipidomic mechanisms.

**Methods:**

A retrospective cohort of 288 patients with HTGP admitted between 2020 and 2025 was included. Patients were categorized into mild acute pancreatitis (MAP) and moderately severe/severe acute pancreatitis (MSAP/SAP). Ensemble feature selection integrating eight algorithms was applied to identify robust predictors. Ten machine learning models were constructed and compared. SHAP (SHapley Additive exPlanations) analysis was used to interpret model outputs. Untargeted serum lipidomics combined with KEGG pathway enrichment analysis was performed in 15 matched severe/non-severe AP pairs to explore the biological relevance of identified predictors.

**Results:**

Among 288 patients, 150 (52.1%) were classified as MSAP/SAP at hospital admission. Ten predictors associated with inflammatory activation, coagulation dysfunction, endothelial leakage, and systemic injury were identified, including IL-6, D-dimer, PCT, albumin, CRP, SIRS, pleural effusion, and pancreatitis-associated ascitic fluid. Among all candidate algorithms, the Naive Bayes model achieved the best overall discrimination (AUC = 0.841), outperforming APACHE II and modified Marshall scores. Calibration assessment revealed a calibration intercept of −0.0332 and a Brier score of 0.2035; the calibration slope of 0.2009 indicated compression of predicted probabilities, suggesting caution in absolute risk estimation. Decision curve analysis demonstrated positive net clinical benefit across threshold probabilities from 0 to approximately 0.85, with the Naive Bayes model outperforming both benchmark scoring systems across clinically relevant thresholds. SHAP analysis identified IL-6, D-dimer, and PCT as the most influential predictors. Exploratory lipidomic pathway enrichment in a sub-cohort of 15 matched pairs identified alterations in glycerophospholipid metabolism, sphingolipid metabolism, necroptosis, and autophagy pathways, providing hypothesis-generating biological context for the identified clinical predictors.

**Conclusion:**

We developed an explainable machine learning framework integrating clinical and exploratory lipidomic evidence for early severity stratification of HTGP at hospital admission. The identified predictors converged on interconnected biological axes involving inflammation, coagulation, and endothelial dysfunction. The lipidomic sub-study provided complementary pathway-level context supporting the biological plausibility of these predictors. As this study is based on a single-center retrospective cohort without external validation, findings should be regarded as preliminary; prospective multicenter validation is required before clinical deployment.

## 1 Introduction

Acute pancreatitis (AP) is a common gastrointestinal emergency with highly heterogeneous clinical manifestations and outcomes. Gallstones, alcohol consumption, and hypertriglyceridemia (HTG) represent the major etiologies of AP worldwide (1, 2). In recent years, accompanied by changes in dietary patterns, increasing prevalence of obesity and metabolic syndrome, and rapid socioeconomic development, the incidence of hypertriglyceridemia-induced pancreatitis, also termed hypertriglyceridemic pancreatitis (HTGP), has risen substantially (1, 3). Current epidemiological evidence indicates that HTGP has become the third leading cause of AP globally (1, 4) and the second most common etiology in China (5, 6). Notably, several studies have reported that HTGP accounts for nearly 40% of severe acute pancreatitis (SAP) cases and is increasingly emerging as the predominant cause of AP among young male patients (2, 7).

Compared with other etiological subtypes, HTGP is characterized by earlier disease onset, higher recurrence rates, more pronounced metabolic dysregulation, and an increased propensity for severe systemic complications (3, 8). HTGP patients are more likely to develop pancreatic necrosis, systemic inflammatory response syndrome (SIRS), multiple organ failure (MOF), and persistent organ dysfunction, all of which contribute to prolonged hospitalization, increased healthcare burden, and elevated mortality risk (1, 5). A multicenter study demonstrated that patients with HTGP exhibited a significantly higher risk of progression to SAP and organ failure compared with non-HTGP populations (OR: 1.897, 95% CI: 1.380 ~ 2.608, *p* < 0.001) (9). Importantly, disease deterioration in HTGP often occurs rapidly during the early stage following admission, highlighting the critical importance of timely risk stratification and early intervention.

Currently, conventional clinical scoring systems, including Ranson, Bedside Index for Severity in Acute Pancreatitis (BISAP), Acute Physiology and Chronic Health Evaluation II (APACHE II), and the modified Marshall score, remain widely used for severity assessment in AP. However, these models were primarily developed for general AP populations and may not adequately capture the unique metabolic-inflammatory characteristics and complex pathophysiological heterogeneity of HTGP (10). In addition, traditional statistical models and scoring systems are limited in their ability to handle nonlinear interactions and high-dimensional clinical variables, resulting in suboptimal predictive performance for early severe progression in HTGP patients (10, 11).

Machine learning (ML) approaches have demonstrated substantial advantages in processing complex multidimensional clinical data, identifying nonlinear associations, and improving predictive discrimination. Recent studies have increasingly applied ML algorithms to disease risk prediction and clinical decision support in critical care and gastrointestinal diseases (11, 12). Nevertheless, most existing prediction models for HTGP remain purely data-driven and primarily focus on post-hoc disease characterization, rather than enabling systematic severity stratification at the time of hospital admission. Moreover, many previously reported ML models lack mechanistic interpretability and rely on single feature-selection strategies, thereby limiting model robustness, biological plausibility, and clinical translatability.

Emerging evidence suggests that HTGP is fundamentally a lipid metabolism-associated inflammatory disease involving dysregulated lipid signaling, endothelial injury, coagulation activation, mitochondrial dysfunction, and inflammatory amplification (3, 5). Therefore, integrating lipidomic evidence with explainable artificial intelligence may provide biologically grounded risk stratification and enhance mechanistic interpretability beyond conventional “black-box” prediction models. Untargeted lipidomic profiling enables systematic characterization of lipid metabolic alterations and may help elucidate the molecular pathways underlying disease severity in HTGP.

Therefore, in the present study, we developed an explainable machine learning framework integrating ensemble feature selection, multi-model machine learning comparison, SHAP (SHapley Additive exPlanations)-based interpretability analysis, and serum lipidomic pathway profiling to construct an early severity stratification model for HTGP. All predictor variables were ascertained within 24 h of hospital admission. The primary clinical objective was to accurately discriminate patients with MSAP/SAP from those with MAP at the time of admission, rather than to predict longitudinal disease progression from a confirmed mild baseline. By combining routinely available clinical indicators with lipidomic pathway evidence, we aimed not only to improve discriminative performance, but also to provide mechanistic insights into the biological processes driving disease severity, thereby facilitating precision risk stratification and timely clinical intervention in HTGP patients.

## 2 Materials and methods

### 2.1 Study design and population

The study is a single-center retrospective cohort study with a cross-sectional severity stratification design. The primary objective was to develop a model for early severity identification of HTGP within 24 h of hospital admission, distinguishing patients with MSAP/SAP from those with MAP. All candidate predictor variables were ascertained within this 24-h landmark window. Severity classification was determined according to the Revised Atlanta Classification based on each patient’s clinical course during the index hospitalization; patients in the MSAP/SAP group may have met the relevant diagnostic criteria at or shortly after admission. This design reflects the real-world clinical scenario in which rapid severity stratification at presentation is critical for early triage and intervention decisions. Patients admitted to the Department of Hepatobiliary Surgery at the General Hospital of Ningxia Medical University between October 1, 2020, and May 30, 2025, were selected as the study subjects.

The inclusion criteria were as follows: (1) First, meeting the diagnostic criteria for acute pancreatitis (AP) as stipulated in the “Chinese Guidelines for the Diagnosis and Treatment of Acute Pancreatitis (2021)”: (a) persistent upper abdominal pain; (b) serum amylase and/or lipase concentrations at least three times the upper limit of normal; (c) abdominal imaging findings consistent with the imaging changes of acute pancreatitis. AP is diagnosed if at least two of the above three criteria are met (13); (2) serum triglyceride (TG) level ≥1,000 mg/dL (11.3 mmol/L); or serum TG level between 5.65–11.3 mmol/L with milky serum appearance (14); (3) age >18 years with complete clinical data.

**Figure 1:**
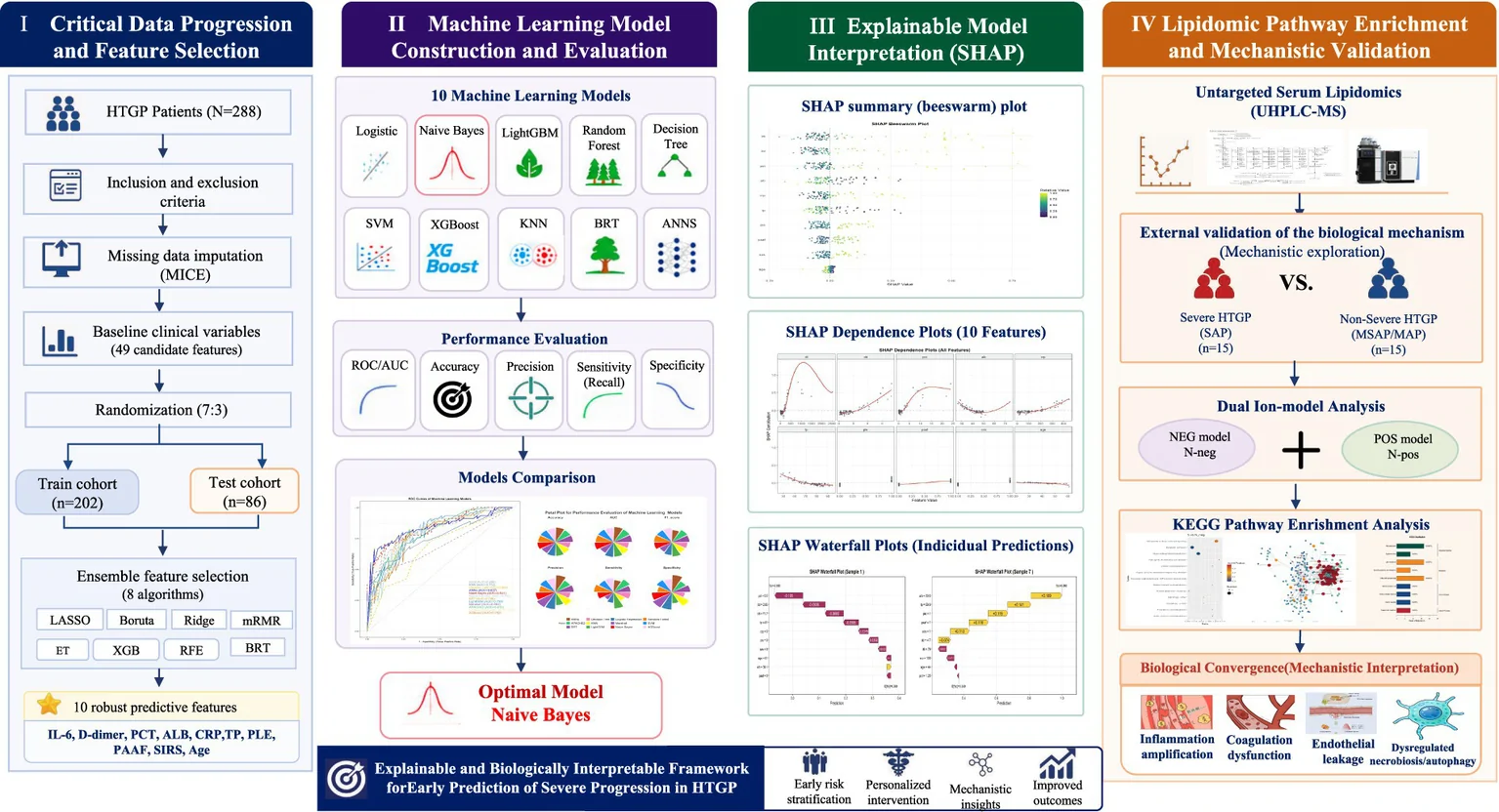
Integrated workflow of explainable machine learning and lipidomic pathway analysis for early severity stratification in HTGP. This study included patient enrollment, clinical data collection, feature selection using eight ensemble algorithms, machine learning model construction and evaluation, SHAP-based interpretability analysis, and serum lipidomic KEGG pathway enrichment analysis. A total of 288 HTGP patients were retrospectively enrolled and randomly divided into training and testing cohorts at a ratio of 7:3.

The exclusion criteria were: (1) presence of factors that could bias the early severe progression of the disease, such as severe heart, lung, liver, kidney, or psychiatric diseases, cardiac function >III, acute myocardial infarction, chronic kidney disease with creatinine clearance rate <40 mL/min, COPD patients requiring long-term oxygen therapy, chronic liver disease with Child-Pugh C liver function, immune diseases or immunosuppressive states, or moribund patients; (2) concurrent tumors of the biliary and pancreatic systems; (3) AP caused by other etiologies such as gallstones or alcohol. A total of 288 study subjects meeting the inclusion and exclusion criteria were ultimately included. The study flowchart is shown in Figure 1.

### 2.2 Selection of study variables

A total of 49 variables were included in this study, consisting of the following categories: general demographic data (age, sex, BMI); underlying comorbidities (overweight, hypertension, diabetes, fatty liver); preoperative laboratory parameters [triglyceride (TG, mmol/l), blood glucose (Glu, mmol/l), amylase (Amy, u/l), lactate (Lac, mmol/l), white blood cell count (WBC, 10^9^/l), platelet count (PLT, 10^9^/l), total cholesterol (TC, mmol/l), Ca^2+^(mmol/l), C-reactive protein (CRP, mg/l), interleukin-6 (IL-6, pg./ml), procalcitonin (PCT, ng/ml), D-dimer (DD, ug/ml), total bilirubin (TB, umol/l), unconjugated bilirubin (UCB, umol/l), total protein (TP, g/l), albumin (ALB, g/l), aspartate aminotransferase (AST, u/l), alanine aminotransferase (ALT, u/l), alkaline phosphatase (ALP, u/l), gamma-glutamyl transferase (GGT, u/l), cholinesterase (CHE, u/l), creatine kinase (CK, u/l), lactate dehydrogenase (LDH, u/l), lipase (LIP, u/l), prothrombin time (PT, s), prothrombin activity (PTA, %), activated partial thromboplastin time (APTT, s), fibrinogen (FIB, g/l), international normalized ratio (INR)]; local pancreatic complications [acute peripancreatic fluid collection (APFC), acute necrotic collection (ANC), pancreatic parenchymal hemorrhage (PPH)]; systemic complications [pleural effusion (PLE), pancreatitis-associated ascitic fluid (PAAF), pelvic effusion (PEE), systemic inflammatory response syndrome (SIRS), multiple organ dysfunction syndrome (MODS), pneumonia, hepatic insufficiency (HI), acute kidney injury (AKI), metabolic acidosis (MA), sepsis]; as well as APACHE II and Marshall scores. All data were collected within 24 h after admission and extracted from the hospital’s electronic medical record system.

### 2.3 Outcome assessment

The outcome of interest in this study is severity classification at hospital admission, distinguishing patients with moderately severe or severe acute pancreatitis (MSAP/SAP) from those with mild acute pancreatitis (MAP), assessed according to the Revised Atlanta Classification (RAC). It is important to note that this study employed a cross-sectional severity stratification design: patients were classified based on their clinical status during the index hospitalization, and a substantial proportion of patients in the MSAP/SAP group had already met the relevant diagnostic criteria at or shortly after the time of admission. The model is therefore intended to support early severity identification within 24 h of admission, rather than to predict longitudinal progression from a confirmed mild baseline. Moderately severe acute pancreatitis (MSAP) is defined as the presence of transient organ failure (≤48 h) and/or local complications. Severe acute pancreatitis (SAP) is defined as the presence of persistent organ failure (>48 h). Organ failure is determined using the modified Marshall scoring system, where a score of ≥2 in any of the three organ systems (respiratory, cardiovascular, or renal) indicates organ failure (15, 16).

### 2.4 Data preprocessing

Due to the retrospective nature of data collection, missing values were present in 25 variables (missing proportion ranging from 0.3 to 33.68%), with 8 variables (IL-6, LDH, CK, Lac, CRP, Ca^2+^, PCT, LIP) exhibiting a missing proportion exceeding 10%. To mitigate uncertainty and the risk of false positives arising from missing data, missing values were addressed using the Multivariate Imputation by Chained Equations (MICE) method (17). The imputation method employed was predictive mean matching (PMM), which preserves the original data distribution and avoids generating unrealistic extreme values. Five imputed datasets were generated, with 10 iterations per dataset, and convergence diagnostics were visually inspected to ensure imputation stability. The final analysis combined results across the multiple imputed datasets to minimize statistical bias inherent in single imputation, adhering to established practices for machine learning and clinical prediction model development. Previous research has demonstrated that MICE outperforms other imputation methods when the proportion of missing data is below 30% (18).

### 2.5 Data set partitioning and model construction

The complete dataset was randomly partitioned into a training set (*n* = 202) and a test set (*n* = 86) at a 7:3 ratio. The training set was used for feature selection, model development, and SHAP analysis, while the test set was reserved exclusively for model evaluation.

### 2.6 Feature selection

To mitigate potential multicollinearity and overfitting risks, enhance the robustness and generalizability of variable selection, and reduce selection bias and overfitting associated with single-method approaches, this study employed eight distinct variable selection methods to identify the optimal subset of features within the training dataset. This ensemble approach aligns with current research trends that favor combining multiple variable selection techniques for feature screening (19, 20). The methods utilized include Least Absolute Shrinkage and Selection Operator (LASSO), Ridge Regression, Elastic Net (ET), Boosted Regression Tree (BRT), eXtreme Gradient Boosting (XG Boost), Maximum Relevance Minimum Redundancy (mRMR), Recursive Feature Elimination (RFE), and Boruta.

### 2.7 Model construction and evaluation

10 machine learning models—Logistic Regression (LR), Light Gradient Boosting Machine (Light GBM), Random Forest (RF), Support Vector Machines (SVM), Naive Bayes (NB), Decision Trees (DT), Artificial Neural Networks (ANNs), K-Nearest Neighbors (KNN), eXtreme Gradient Boosting (XGBoost), and Boosted Regression Tree (BRT)—were trained on the training dataset and subsequently evaluated on the independent test dataset to assess their predictive performance and robustness. Model performance was comprehensively evaluated using multiple metrics, including Area Under the Curve (AUC) with 95% Confidence Interval (95% CI), Accuracy, Sensitivity, Precision, F1 score, and Specificity. The Receiver Operating Characteristic (ROC) curve, which plots the True Positive Rate (Sensitivity) against the False Positive Rate (1-Specificity) across various classification thresholds, was used to visualize and compare the overall discriminatory ability of the models. The AUC, derived from the ROC curve, provides a single scalar measure of a model’s ability to distinguish between positive and negative classes, with values closer to 1 indicating superior performance.

### 2.8 Interpretation of the optimal model

SHAP (SHapley Additive exPlanations) values were computed for each predictor variable to determine feature importance, assessing the direction and magnitude of each feature’s impact on the model’s predictions. A positive SHAP value indicates that the feature increases the predicted probability of MSAP/SAP, while a negative SHAP value suggests a reduction in risk. The absolute value of the SHAP value reflects the extent of a feature’s contribution to the model’s predictive performance.

Visualization techniques were used to present the interpretability results. A SHAP summary plot (beehive plot) was generated to display the ranking of important features based on their mean absolute SHAP values. SHAP waterfall plots were utilized to illustrate the contribution of each feature to individual prediction outcomes, showing how specific feature values shift the prediction from the baseline. Additionally, SHAP dependence plots were created to visually depict the relationship between a given feature and its corresponding SHAP values, highlighting the dependency and interaction effects between variables.

### 2.9 Serum lipidomic profiling and KEGG pathway analysis

To further investigate the molecular mechanisms underlying disease severity in HTGP and biologically support the explainable machine learning findings, untargeted serum lipidomic profiling was performed in a matched sub-cohort of HTGP patients. Based on predefined inclusion and exclusion criteria, serum samples from 15 patients with severe progression (SAP) and 15 patients with MAP/MSAP were included for lipidomic analysis.

Peripheral venous blood samples were collected within 24 h after admission following overnight fasting. After centrifugation at 3000 rpm for 10 min at 4 °C, serum samples were aliquoted and stored at −80 °C until further analysis.

Untargeted lipidomic profiling was conducted using ultra-high-performance liquid chromatography coupled with mass spectrometry (UHPLC–MS) under both positive-ion (pos) and negative-ion (neg) electrospray ionization modes. Raw mass spectrometry data were processed for peak extraction, alignment, and normalization prior to downstream analysis to minimize technical variation.

Differential lipid metabolites between the severe progression group and non-severe AP group were subsequently subjected to Kyoto Encyclopedia of Genes and Genomes (KEGG) pathway enrichment analysis to identify major metabolic pathways associated with severe progression in HTGP. KEGG enrichment results obtained from both POS and NEG ion modes were visualized using bubble plots, with bubble size representing the number of enriched metabolites and color intensity indicating enrichment significance.

To further characterize the biological relationships among altered lipid metabolic pathways and associated metabolites, pathway-metabolite interaction networks were constructed based on KEGG enrichment results. In addition, KEGG classification analysis was performed to evaluate the distribution characteristics of major metabolite subclasses associated with severe progression.

This integrated lipidomic analysis was designed to complement the explainable machine learning framework and provide molecular-level evidence supporting the biological plausibility of the identified clinical predictors.

It should be noted that the grouping strategy used in the lipidomic analysis differed from that applied in the machine learning model construction. The machine learning framework was designed for early clinical risk stratification; therefore, patients were categorized into MAP and MSAP/SAP groups to maximize sensitivity for early identification of non-mild disease progression. In contrast, the lipidomic analysis aimed to explore the biological mechanisms underlying severe disease deterioration. Accordingly, an extreme-phenotype strategy was adopted by comparing SAP patients with MAP/MSAP patients, thereby enhancing the detection of biologically relevant metabolic alterations associated with critical disease severity.

### 2.10 Statistical methods

Data entry and analysis were performed using SPSS 29.0 (IBM). The normality of continuous variables was assessed using the Shapiro–Wilk test. Normally distributed data are presented as mean ± standard deviation (SD), and intergroup comparisons were conducted using the independent samples t-test. Non-normally distributed data are presented as median (interquartile range), and intergroup comparisons were conducted using the Wilcoxon rank-sum test. Categorical variables are presented as frequency (%), and intergroup comparisons were conducted using the *χ^2^* test or *Fisher’s* exact test. A two-tailed *p*-value < 0.05 was considered statistically significant. Feature selection, model construction and evaluation, and SHAP interpretability analysis were performed using R 4.5.2 software.

## 3 Results

### 3.1 General clinical characteristics of the study population

The overall study workflow is illustrated in Figure 1. A total of 288 patients with HTGP were retrospectively enrolled, including 222 males (77.08%). Among them, 150 patients (52.08%) progressed to moderately severe or severe acute pancreatitis (MSAP/SAP), while 138 patients remained in the MAP group. Comparative analysis between the two groups demonstrated significant differences in multiple clinical and laboratory parameters associated with inflammatory activation, coagulation dysfunction, and systemic injury. Specifically, serum Ca^2+^, CRP, IL-6, PCT, D-dimer, TP, ALB, ALP, SIRS, sepsis incidence, APACHE II score, and modified Marshall score differed significantly between the two groups (all *p* < 0.001). Detailed baseline characteristics are summarized in Table 1.

**Table 1:** Description of baseline characteristics of study subjects (*N* = 288, %).

Variable		MAP [*n* = 138]	MSAP/SAP [*n* = 150]	Statistics	*p*-value
Age		37.80 ± 9.404	37.26 ± 9.283	0.238	0.626
Sex	Male	104 (75.36)	118 (78.67)	0.444	0.505
	Female	34 (24.64)	32 (21.33)		
BMI		26.91 ± 3.315	27.46 ± 4.37	1.453	0.229
Over Weight		110 (79.71)	119 (79.33)	0.006	0.937
Hypertension		24 (17.39)	42 (28.00)	4.579	0.032
Diabetes (DM)		52 (37.68)	69 (46.0)	2.042	0.153
Fat Liver (FL)		107 (77.54)	113 (75.33)	0.193	0.660
Triglyceride (TG)		14.39 [6.39, 25.97]	15.4 [6.08, 31.89]	−1.227	0.220
Blood glucose (Glu)		9.42 [7.06, 14.55]	12.63 [8.42, 18.50]	−3.165	0.002
Blood amylase (Amy)		233.45 [111.50, 386.75]	250.05 [107.90, 476.35]	−0.918	0.359
Lactate (Lac)		1.60 [1.14, 2.41]	1.91 [1.32, 3.01]	−2.903	0.004
White blood cell (WBC)		13.11 ± 4.470	14.14 ± 5.562	2.933	0.088
Platelet (PLT)		253.81 ± 80.56	233.54 ± 85.791	4.253	0.040
Total Cholesterol (TC)		6.48 [5.69, 8.76]	7.03 [5.34, 10.85]	−1.050	0.294
Ca^2+^		2.25 [1.92, 2.36]	1.99 [1.24, 2.23]	−4.510	<0.001^*^
C-reactive protein(CRP)		90 [32.70, 162.25]	138 [83.95, 228.25]	−4.267	<0.001^*^
Interleukin-6 (IL-6)		40.40 [14.76, 111.55]	110.00 [43.70, 203.40]	−5.494	<0.001^*^
Procalcitonin (PCT)		0.46 [0.16, 0.72]	0.87 [0.43, 2.25]	−6.261	<0.001^*^
D-dimer (DD)		0.90 [0.38, 1.82]	1.74 [0.79, 4.80]	−5.521	<0.001^*^
Total Bilirubin (TB)		18.80 [14.08, 26.50]	21.05 [15.90, 30.30]	−2.567	0.01
Unconjugated bilirubin (UCB)		14.07 ± 7.558	15.08 ± 8.527	1.136	0.287
Total protein (TP)		78.80 [73.46, 83.00]	73.15 [62.45, 79.75]	−4.987	<0.001^*^
Albumin (ALB)		44.15 [40.10, 47.53]	39.55 [34.00, 43.83]	−6.413	<0.001^*^
Aspartate aminotransferase (AST)		36.44 ± 43.79	47.32 ± 48.53	3.963	0.047
Alanine aminotrans ferase (ALT)		43.82 ± 28.21	60.31 ± 170.68	1.257	0.256
Alkaline phosphatase (ALP)		88.05 [72.65, 106.15]	87.35 [69.65, 111.90]	−0.371	0.711
Gamma-glutamyl transferase (GGT)		58.95 [38.25, 119.98]	62.00 [36.98, 132.75]	−0.306	0.760
Cholinesterase (CHE)		10, 175 [8891.50, 11265.5]	9, 188 [7296.75, 11182.75]	−3.408	<0.001^*^
Creatine kinase (CK)		78.15 [53.63, 140.65]	90.80 [55.33, 140.65]	−1.550	0.121
Lactate dehydrogenase (LDH)		327.50 [224.00, 442.00]	389.00 [248.75, 734.50]	−3.389	<0.001^*^
Lipase (LIP)		1789.00 [654.75, 4607.00]	1546.50 [535.25, 5148.25]	−0.368	0.713
Prothrombin time (PT)		10.90 [10.40, 11.63]	11.60 [10.60, 12.60]	−4.051	<0.001^*^
Prothrombin activity (PTA)		102.90 ± 16.53	94.74 ± 18.70	15.288	<0.001^*^
Activated partial thromboplastin time (APTT)		30.39 ± 4.17	31.05 ± 6.15	1.119	0.291
Fibrinogen (FIB)		4.22 ± 1.95	4.82 ± 2.20	5.978	0.015
International normalized ratio (INR)		0.99[0.94, 1.05]	1.04[0.95, 1.13]	−3.744	<0.001^*^
Acute peripancreatic fluid accumulation (APFC)		134 (97.10)	149 (99.33)	2.099	0.147
Acute necrotic collection (ANC)		14 (10.14)	43 (28.67)	15.532	<0.001^*^
Pancreatic parenchymal hemorrhage (PPH)		0 (0.00)	4 (2.67)	3.732	0.053
Pleural effusion (PLE)		12 (8.70)	64 (42.67)	42.70	<0.001^*^
Pancreatitis associated ascites fluid (PAAF)		23 (16.67)	80 (53.33)	42.06	<0.001^*^
Pelvic effusion (PEE)		24 (17.39)	58 (38.67)	15.975	<0.001^*^
Systemic inflammatory response syndrome (SIRS)		11 (7.97)	59 (39.33)	38.426	<0.001^*^
Multiple organ dysfunction syndrome (MODS)		0 (0.00)	21 (14.00)	20.84	<0.001^*^
Pneumonia		29 (21.01)	68 (45.33)	19.030	<0.001^*^
Hepatic insufficiency (HI)		2 (1.45)	18 (12.00)	12.381	<0.001^*^
Acute kidney injury (AKI)		3 (2.17)	18 (12.00)	10.266	0.001^*^
Metabolic acidosis (MA)		1 (0.72)	20 (13.33)	16.903	<0.001^*^
Sepsis		3 (2.17)	21 (14.00)	13.159	<0.001^*^
APACHE II		3.00 [2.00, 6.00]	7.00 [4.00, 10.00]	−7.251	<0.001^*^
Marshall		0.00 [0.00, 1.00]	1.00 [0.00, 2.00]	−5.740	<0.001^*^

*p <0.001.

### 3.2 Ensemble feature selection for identification of robust predictive variables

**Figure 2:**
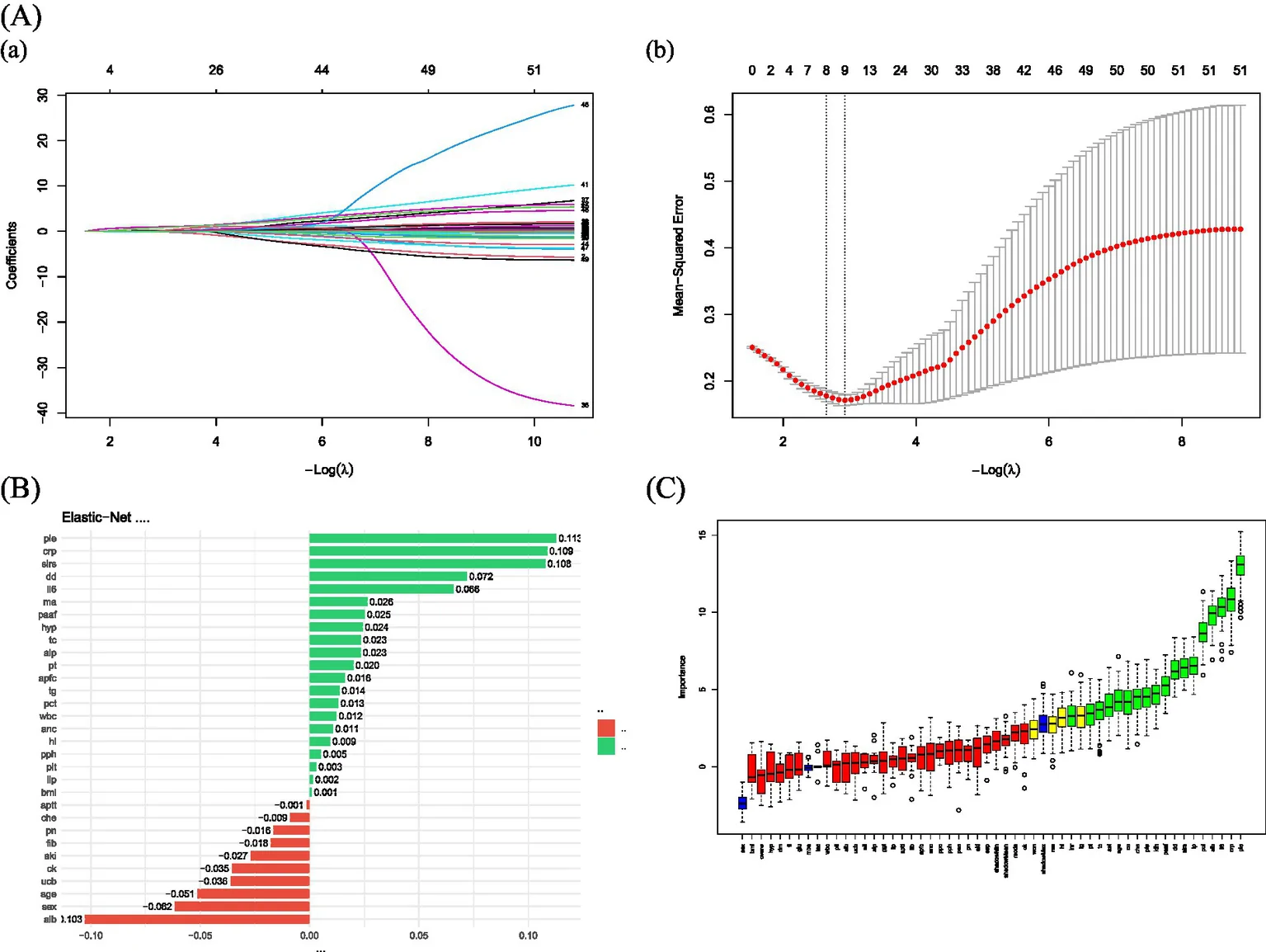
Ensemble feature selection process for identifying robust predictors associated with severity stratification in HTGP. **(A)** LASSO regression coefficient profiles of candidate variables **(a)** Coefficient trajectory plot of LASSO regression; **(b)** Cross- validation curve and optimal parameter selection of LASSO regression. **(B)** Elastic-Net regression coefficient plot showing positive and negative regression coefficients of all candidate variables. **(C)** Boruta feature selection analysis illustrating the importance ranking of candidate predictors. Variables selected by at least three feature selection algorithms were retained as robust predictive features for subsequent machine learning model construction. LASSO, least absolute shrinkage and selection operator.

To identify robust predictors associated with early severity stratification in HTGP, eight machine learning-based feature selection algorithms were integrated to perform ensemble feature screening using the training dataset (Figure 2). Since APACHE II and modified Marshall scores were predefined clinical scoring systems, they were excluded from the feature selection procedure to avoid introducing composite-score bias into model construction.

The LASSO regression model initially identified eight candidate variables and served as the baseline reference for subsequent feature integration. For each feature selection algorithm, approximately 8–12 variables with the highest importance rankings were retained for downstream analysis. Variables selected by at least three independent algorithms were ultimately defined as robust predictive features associated with progression to MSAP/SAP in HTGP patients.

**Figure 3:**
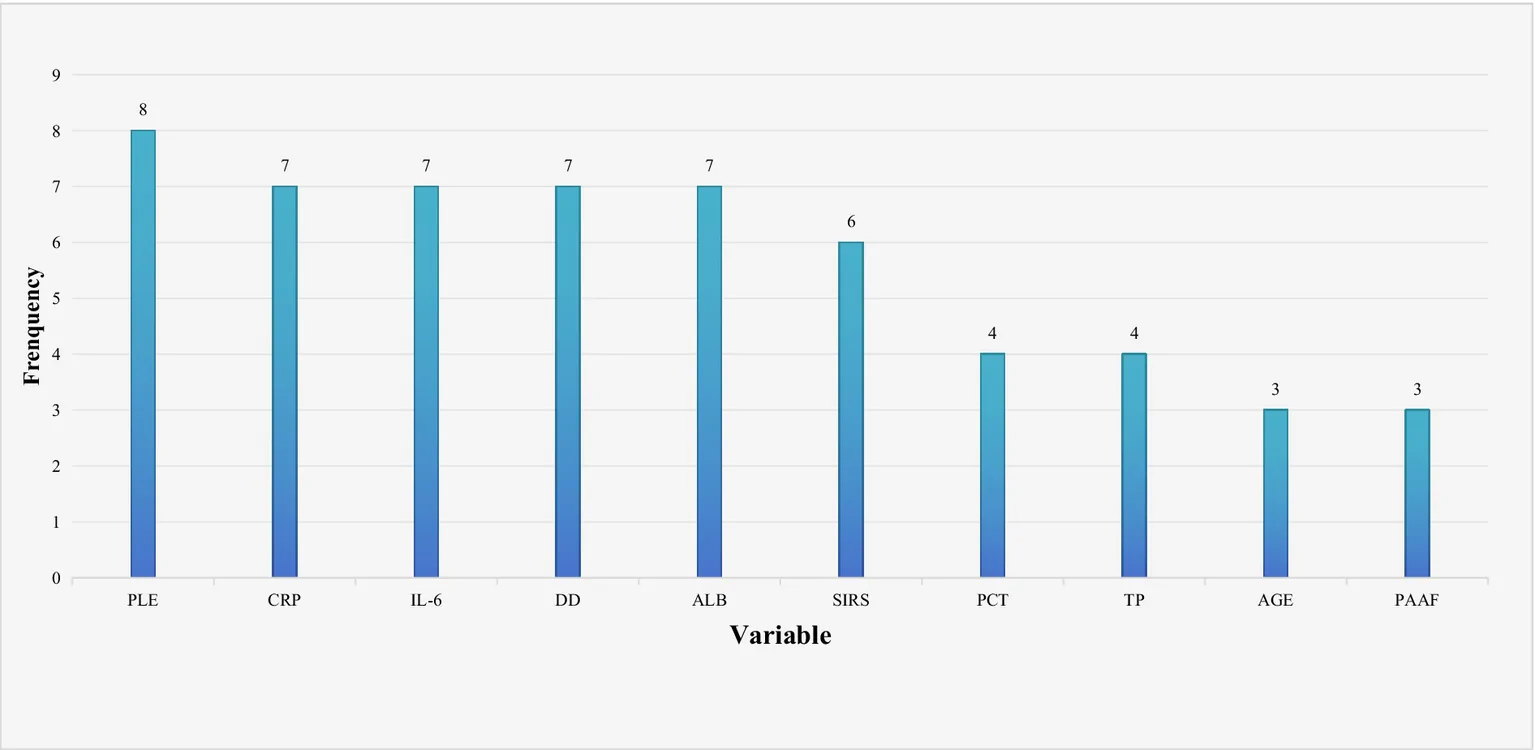
Integrated visualization of the selected predictive features associated with Predictors stratification for MSAP/SAP in HTGP. Ten robust predictors were ultimately identified through ensemble feature selection, including inflammatory markers, coagulation indicators, protein metabolism markers, and systemic inflammatory response-related variables. The selected features were subsequently incorporated into machine learning model development. PLE, pleural effusion; CRP, C-reactive protein; IL-6, interleukin-6; DD, D-dimer; ALB, albumin; SIRS, systemic inflammatory response syndrome; PCT, procalcitonin; TP, total protein; PAAF, pancreatitis-associated ascitic fluid.

As shown in Figure 3, ten predictors were finally retained for model construction, including PLE, CRP, IL-6, D-dimer, ALB, SIRS, PCT, TP, age, and PAAF. Subsequent univariate analysis demonstrated that nine of the ten selected variables differed significantly between the two groups (all *p* < 0.001), whereas age showed a borderline association (*p* = 0.067) (Table 2).

**Table 2:** Comparisons of 10 characteristic predictors in the training set (*n* = 202, %).

Variable	MAP (*n* = 97)	MSAP/SAP (*n* = 105)	Statistics	*p* value
Age	38.49 ± 9.77	36.02 ± 9.33	3.395	0.067
C-reactive protein (CRP)	90.0 [33.89, 160.0]	153 [100.0, 255.0]	−4.721	<0.001
Interleukin-6 (IL-6)	34.0 [12.45, 87.45]	111.0 [43.65, 195.50]	−5.171	<0.001
Procalcitonin (PCT)	0.37 [0.16, 0.59]	0.76 [0.42, 1.85]	−5.355	<0.001
D-dimer (DD)	0.84 [0.38, 1.74]	1.59 [0.73, 4.83]	−4.319	<0.001
Total protein (TP)	77.9 [71.55, 82.6]	72.2 [61.55, 78.85]	−4.316	<0.001
Albumin (ALB)	43.2 [40.10, 47.25]	40.4 [34.20, 44.15]	−4.894	<0.001
Pleural effusion (PLE)	7 (7.22)	48 (45.71)	37.714	<0.001
Pancreatitis associated ascites fluid (PAAF)	18 (18.56)	54 (51.43)	23.751	<0.001
Systemic inflammatory response syndrome (SIRS)	9 (9.28)	40 (38.10)	22.789	<0.001

**Figure 4:**
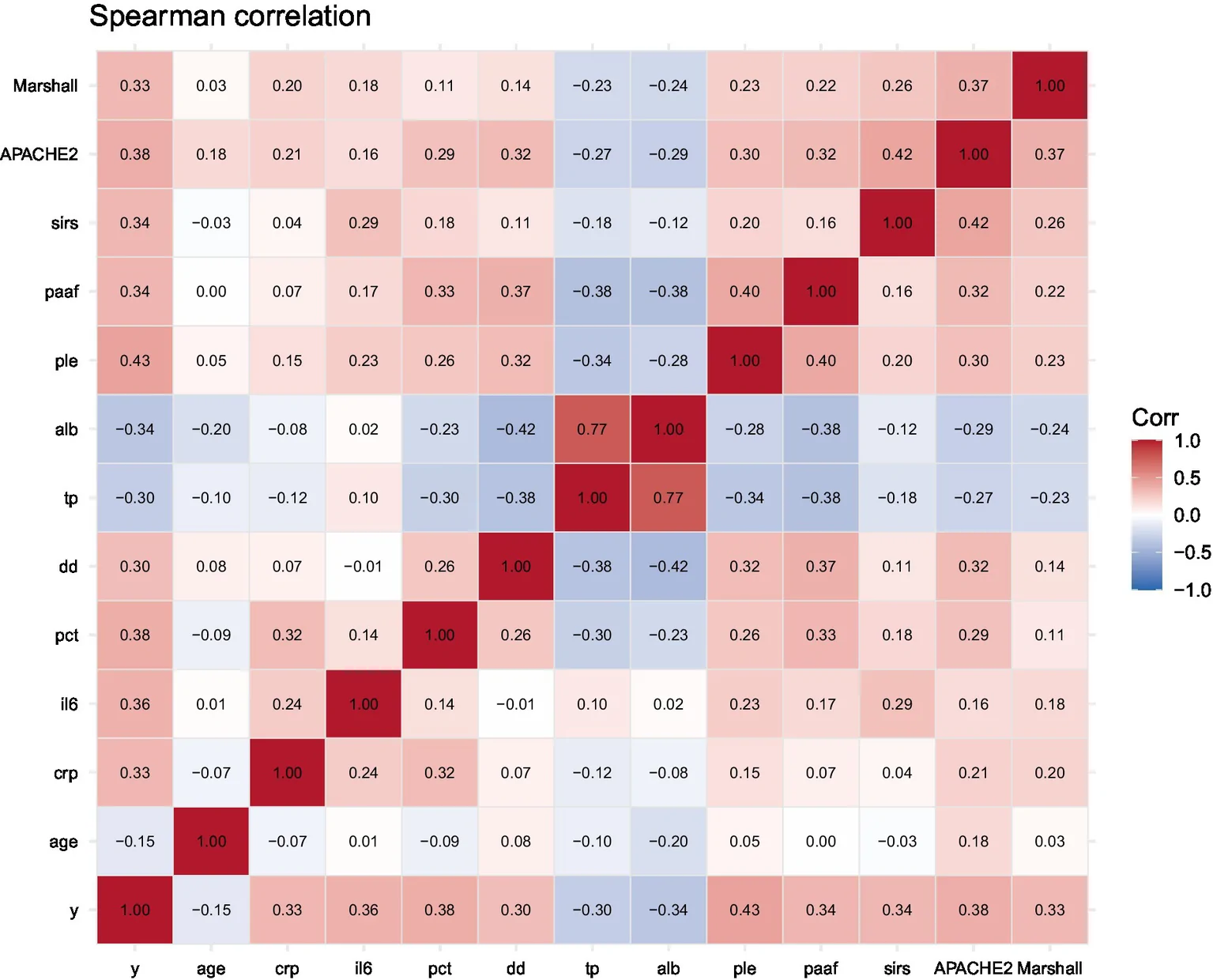
Correlation heatmap of the selected predictive features in the training cohort. Red indicates positive correlation, whereas blue indicates negative correlation. Colo intensity corresponds to the magnitude of the correlation coefficient. The selected predictors demonstrated significant associations with severe progression-related outcomes while maintaining acceptable inter-feature correlations.

Spearman correlation analysis further demonstrated significant associations between the selected predictors and severe outcomes, supporting the potential predictive relevance. The inter-feature correlation structure is visualized in the heatmap presented in Figure 4.

### 3.3 Construction and performance evaluation of machine learning models

A total of ten machine learning algorithms, including XGBoost, logistic regression, LightGBM, random forest, and Naive Bayes, were trained and evaluated using the training dataset to establish an early prediction model for severity stratification in HTGP patients.

**Figure 5:**
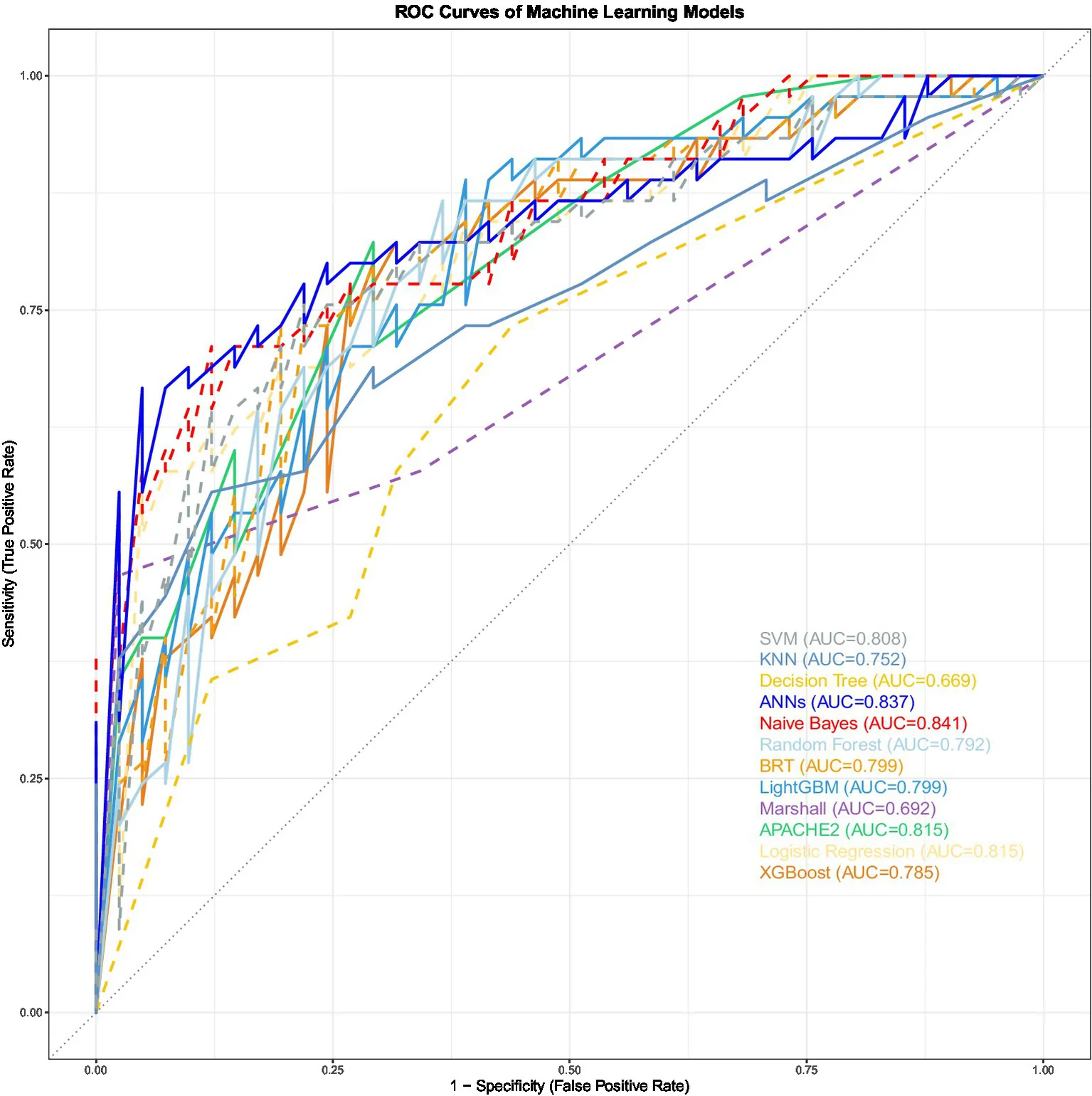
Receiver operating characteristic (ROC) curves of the machine learning models for early stratification of severe progression to MSAP/SAP in HTGP. Ten machine learning modesl were trained and evaluated using the testing cohort. The Naive Bayes model demonstrated the best overall discriminatory performance with the highest area under the curve (AUC), outperforming conventional clinical scoring systems. AUC, area under the curve; ROC, receiver operating characteristic.

**Figure 6:**
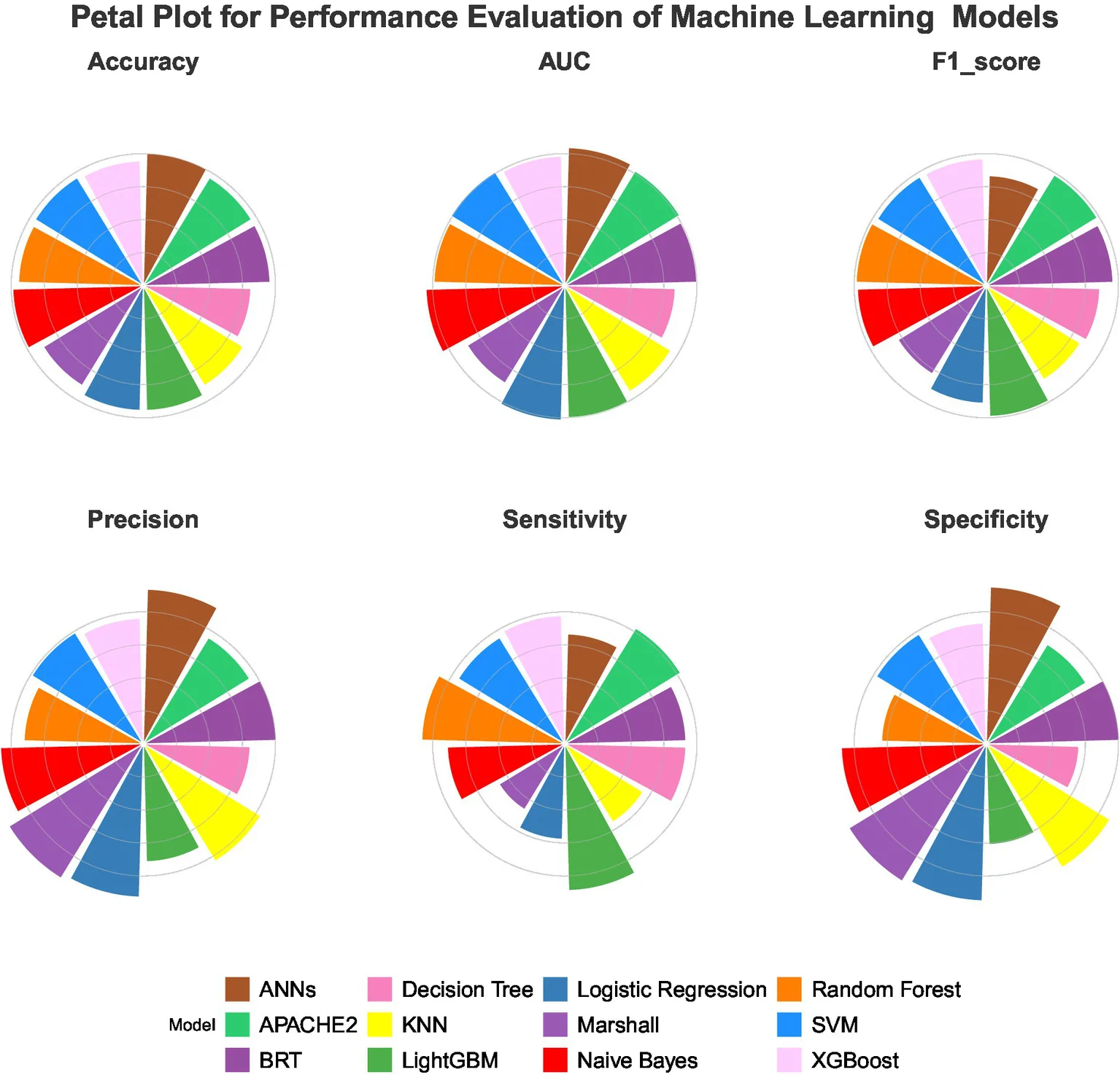
Radar plot comparing predictive performance metrics among different machine learning models. Model performance was comprehensively evaluated based on accuracy, sensitivity, specificity, precision, F1 score, and AUC. The Naive Bayes model achieved the most balanced overall predictive performance among all candidate algorithms.

Among all candidate algorithms, the Naive Bayes model demonstrated the most favorable overall predictive performance, particularly in terms of discriminatory ability and sensitivity for identifying progression to MSAP/SAP (Table 3). Receiver operating characteristic (ROC) curve analysis for all machine learning models is presented in Figure 5, while the comparative radar plot visualization is shown in Figure 6. Of the 10 predictors ultimately identified in our feature selection pipeline, PLE, PAAF and SIRS represent secondary complications that develop as severe pancreatitis progresses. Accordingly, we excluded these three variables to conduct a sensitivity analysis. The resulting model yielded an AUC value of 0.804, which was inferior to the AUC of the original model incorporating all 10 predictors (0.841).

**Table 3:** Comparison of evaluation metrics for machine learning models.

Model	AUC	95% CI	best cut	Accuracy	Sensitivity/Recall	Precision	F1 score	Specificity
XGBoost	0.785	0.688 ~ 0.883	0.528	0.756	0.778	0.761	0.769	0.732
Logistic regression	0.815	0.725 ~ 0.904	0.801	0.756	0.578	0.929	0.712	0.951
LightGBM	0.799	0.707 ~ 0.892	0.080	0.756	0.889	0.714	0.792	0.610
BRT	0.799	0.704 ~ 0.894	0.685	0.767	0.733	0.805	0.767	0.805
Random forest	0.792	0.696 ~ 0.889	0.451	0.756	0.867	0.722	0.788	0.634
Naive Bayes	**0.841**	**0.759 ~ 0.922**	**0.410**	**0.791**	**0.711**	**0.865**	**0.780**	**0.878**
ANNs	0.837	0.750 ~ 0.924	0.876	0.802	0.667	0.938	0.667	0.951
Decision tree	0.669	0.559 ~ 0.779	0.372	0.651	0.733	0.647	0.688	0.561
KNN	0.752	0.649 ~ 0.855	0.654	0.709	0.556	0.833	0.667	0.878
SVM	0.808	0.714 ~ 0.901	0.624	0.767	0.756	0.791	0.773	0.780
APACHEII	0.815	0.728 ~ 0.903	0.480	0.767	0.822	0.755	0.787	0.707
Marshall	0.692	0.59 ~ 0.795	0.666	0.709	0.467	0.955	0.627	0.976

XG Boost: eXtreme Gradient Boosting; BRT: Boosted regression tree; ANNs: Artificial neural networks; KNN: K-Nearest Neighbors; SVM: Support Vector Machines. Bold values in table denote the optimal predictive model.

### 3.4 Naive bayes model performance comparison, calibration, and clinical utility

**Figure 7:**
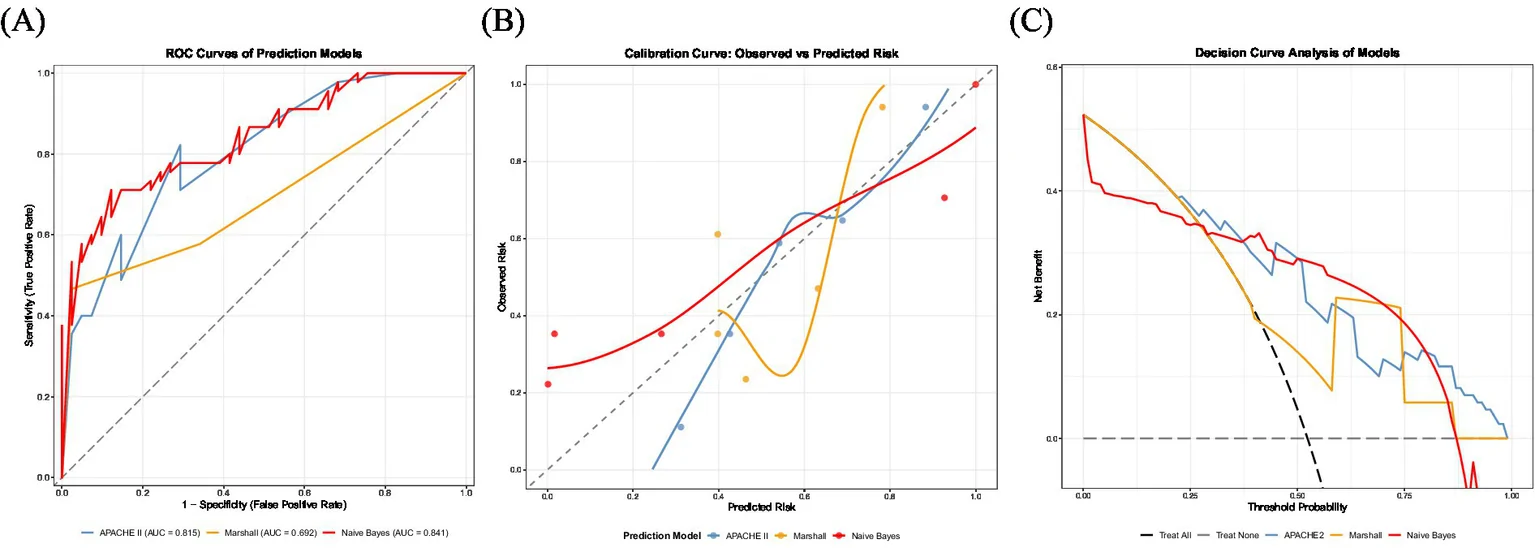
Comparative ROC analysis, calibration curve analysis and decision curve analysis of the Naive Bayes model, APACHE Il score, and modified Marshall score. The explainable machine learning-based Naive Bayes model demonstrated superior predictive discrimination for early to MSAP/SAP in HTGP compared with APACHE II and Marshall scoring systems.

To validate the clinical applicability of the developed model, the optimal Naive Bayes model was compared with two conventional severity assessment systems, namely the APACHE II score and modified Marshall score. ROC curve analysis verified that the Naive Bayes model possessed better discriminative ability for early severity stratification in HTGP patients than the conventional scoring systems (Figure 7A).

The calibration performance of the three models was further evaluated on an independent test set, with visualized calibration curves and quantitative calibration metrics, (Figure 7B and Table 4). The Naive Bayes model presented negligible systematic prediction bias but compressed predicted probabilities relative to actual clinical outcomes, a typical characteristic of Naive Bayes classifiers constrained by the conditional independence assumption. In contrast, the APACHE II and modified Marshall scores exhibited overdispersed predicted probabilities and unstable calibration performance across different probability ranges. Overall probabilistic accuracy was comparable among the three models, as summarized in Table 4. Collectively, despite its superior discriminative power, the Naive Bayes model requires cautious interpretation of absolute probability estimates, and recalibration based on a larger independent cohort is warranted before clinical application.

**Table 4:** Calibration performance of the 3 models on the test set.

Model	Calibration intercept	Calibration slope	Brier score
Naive Bayes	−0.0332	0.2009	0.2035
APACHE II	−0.2997	1.4968	0.1819
Marshall	−0.0675	1.3715	0.2108

Decision curve analysis (DCA) was conducted to assess the net clinical benefit of each model across different threshold probabilities (Figure 7C). The Naive Bayes model yielded sustained positive net benefit over the blank treat-none strategy within a wide range of threshold probabilities. Notably, it achieved the highest net benefit among all competing models at low threshold probabilities corresponding to early clinical intervention scenarios. In the medium threshold range, the Naive Bayes model maintained stable net clinical benefit, while the conventional scoring systems showed obvious fluctuations in net benefit performance.

These results indicate that the Naive Bayes model provides more stable and reliable clinical decision-making benefits for HTGP patient severity stratification within clinically practical risk threshold ranges, demonstrating promising potential as a clinical triage auxiliary tool pending further external validation.

### 3.5 SHAP-based interpretability analysis of the optimal naive bayes model

To address the inherent “black-box” nature of machine learning models, SHAP (21) analysis was employed to interpret the final optimal Naive Bayes model and elucidate its prediction outcomes. To improve interpretability of the optimal prediction model and quantify the contribution of individual predictors, SHAP analysis was performed for the Naive Bayes classifier.

**Figure 8:**
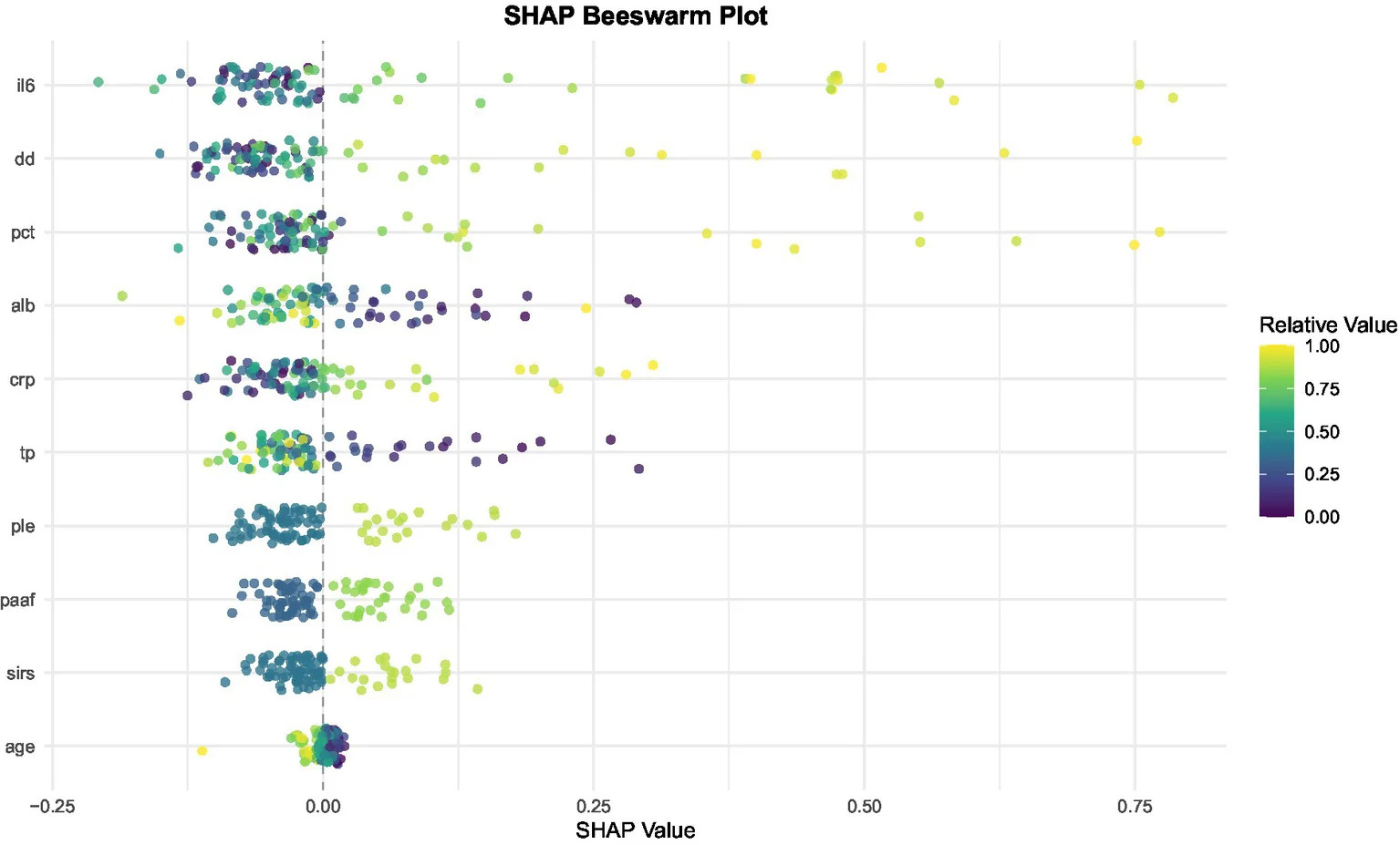
SHAP summary (beeswarm) plot illustrating feature importance in the optimal Naive Bayes model. Features are ranked according to mean absolute SHAP values, reflecting their overall contribution to model prediction. Higher SHAP values indicate a greater contribution to severe progression risk. IL-6, D-dimer, and PCT were identified as the most influential predictors. SHAP, SHapley Additive exPlanations.

The SHAP summary beeswarm plot demonstrated the relative importance distribution of the selected predictive variables (Figure 8). The predictors were ranked according to mean absolute SHAP values as follows: IL-6, D-dimer, PCT, ALB, CRP, TP, PLE, PAAF, SIRS, and age. Among these, IL-6 exhibited the strongest contribution to the model’s discrimination of MSAP/SAP from MAP.

**Figure 9:**
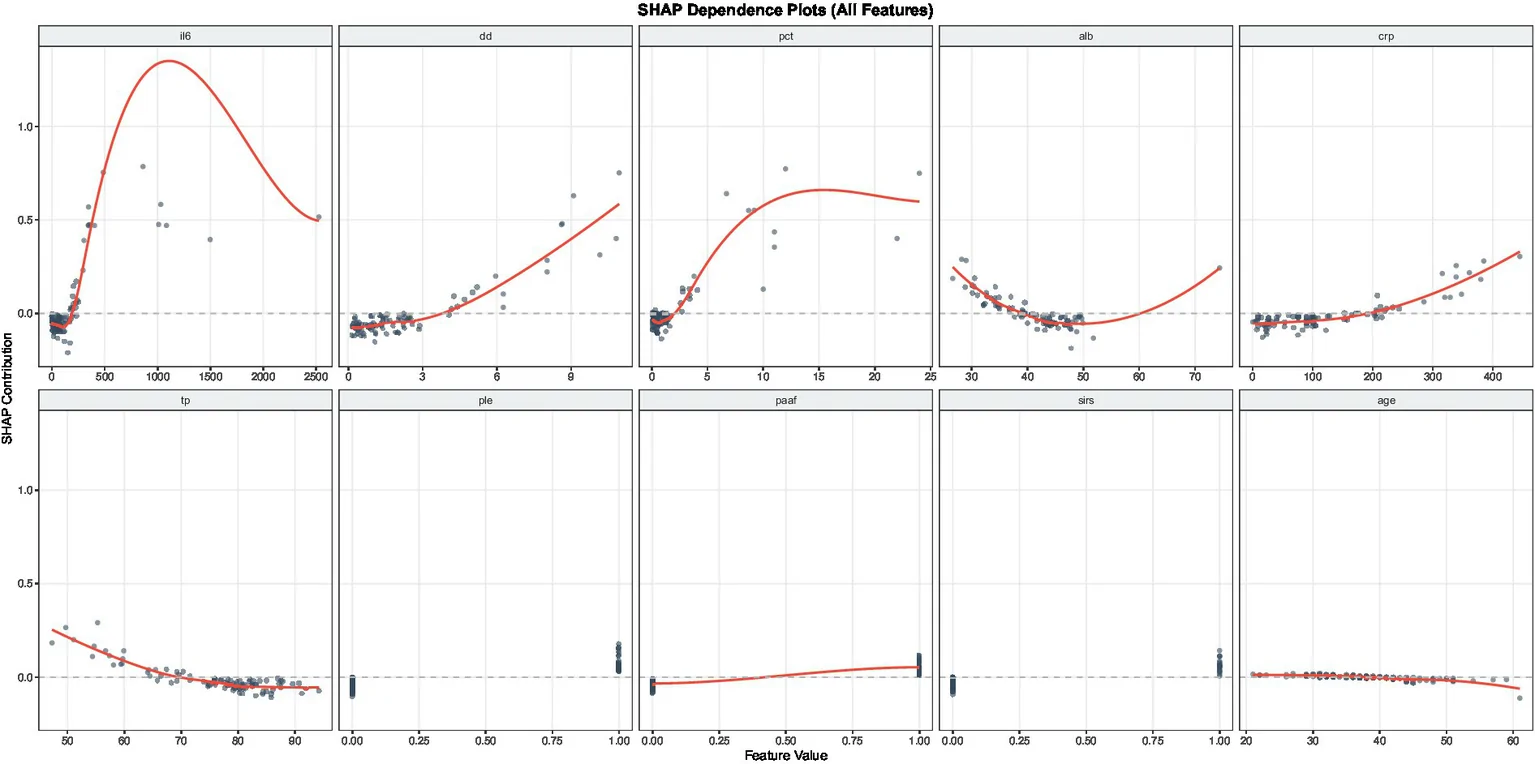
SHAP dependence plots demonstrating the relationship between key predictors and early stratification of severe progression risk. Each dot represents an individual patient. The *x*-axis indicates the feature value, whereas the *y*-axis represents the corresponding SHAP value. Positive SHAP values indicate increased risk of progression to MSAP/SAP.

SHAP dependence analysis further characterized the directional relationship between each predictor and model output (Figure 9). Elevated IL-6, D-dimer, PCT, and CRP levels were positively associated with the predicted probability of MSAP/SAP, indicating that increasing values of these inflammatory and coagulation-related indicators corresponded to higher predicted severity. In contrast, ALB and TP demonstrated negative associations with predicted severity, consistent with their established roles as markers of systemic vascular permeability and plasma protein extravasation. Younger age was also associated with higher predicted severity. The presence of PLE, PAAF, and SIRS was associated with substantially increased predicted probability of MSAP/SAP; as noted in the study design, these variables were ascertained within 24 h of admission and reflect systemic disease burden at presentation rather than antecedent risk factors per se. The SHAP summary beeswarm plot demonstrated the relative importance distribution of the selected predictive variables (Figure 8). The predictors were ranked according to mean absolute SHAP values as follows: IL-6, D-dimer, PCT, ALB, CRP, TP, PLE, PAAF, SIRS, and age. Among these, IL-6 exhibited the strongest contribution to severe progression risk.

To facilitate clinical implementation, optimal cutoff values for continuous predictors were subsequently determined using the Youden index. The identified thresholds were as follows: IL-6192.782 pg./mL, D-dimer 3.807 μg/mL, PCT 2.06 ng/mL, ALB 39.202 g/L, CRP 193.303 mg/L, TP 69.694 g/L, and age 38.858 years. These thresholds provide actionable reference values that may support bedside severity stratification within 24 h of admission, pending external validation.

### 3.6 Lipidomic KEGG pathway enrichment analysis

**Figure 10:**
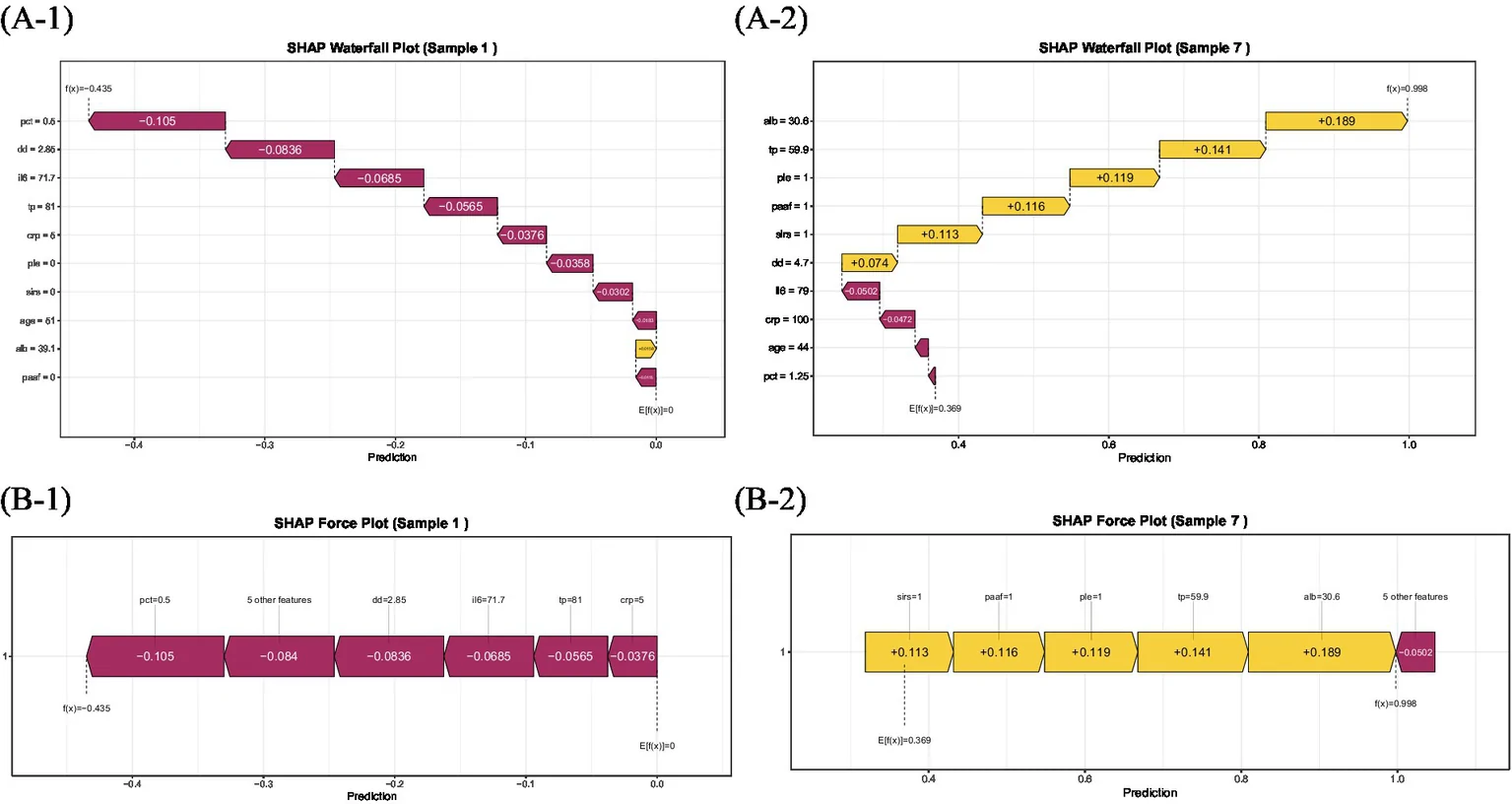
Representative SHAP waterfall plot illustrating individualized prediction interpretation in the Naive Bayes model. The waterfall plot demonstrates how each feature contributes to shifting the prediction probability from the baseline value toward the final prediction outcome in an individual patient. **(a)** SHAP force plot for negative Sample 1 prediction. **(b)** SHAP force plot for positive Sample 7 prediction.

To explore potential lipid metabolic alterations associated with disease severity in HTGP and provide complementary biological context for the machine learning findings, serum lipidomic profiling followed by KEGG pathway enrichment analysis was performed as a hypothesis-generating sub-study in 15 matched pairs of severe and non-mild HTGP patients using both negative-ion (N_neg) and positive-ion (N_pos) detection modes (Figure 10).

**Figure 11:**
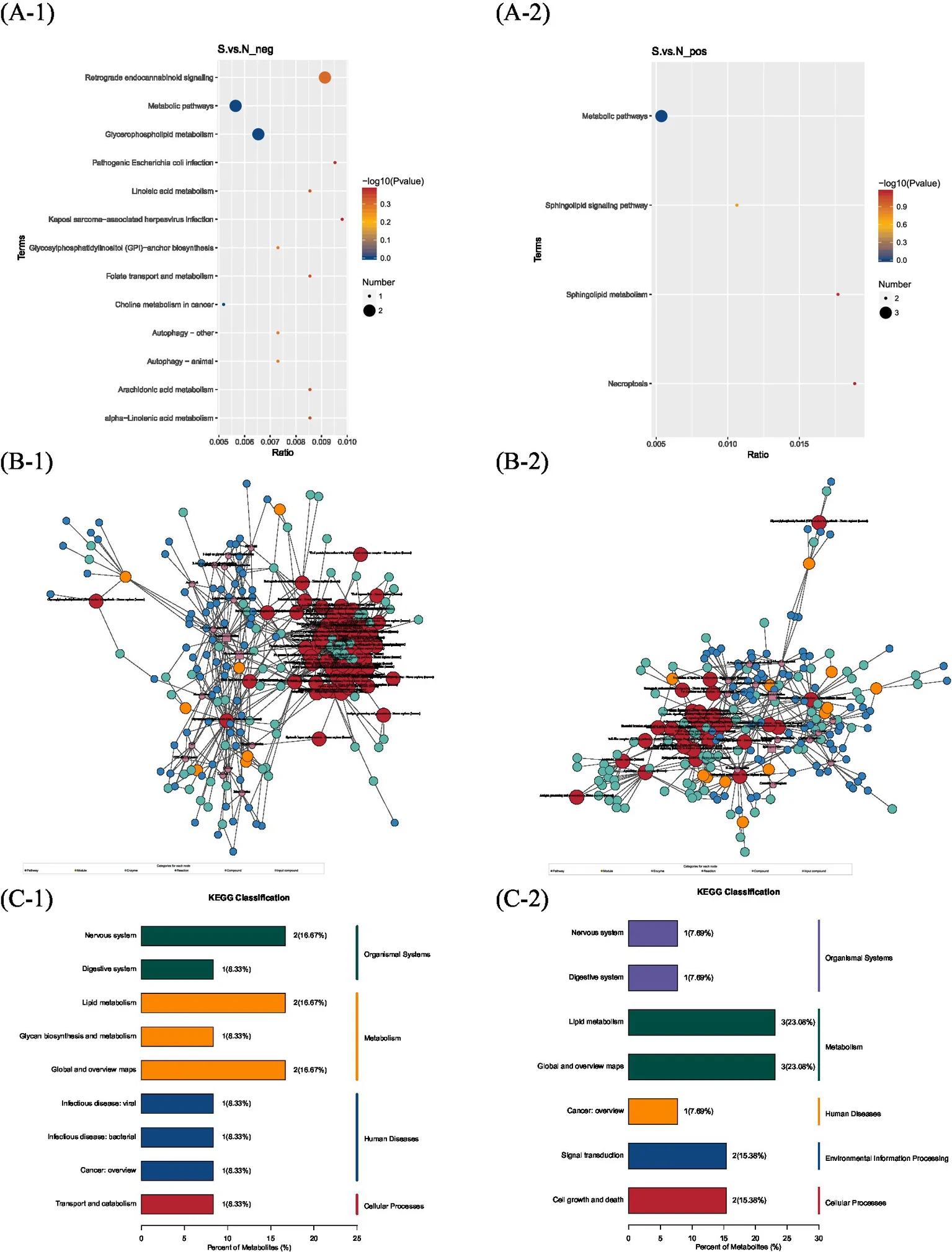
KEGG pathway enrichment analysis of serum lipidomics in severe versus non-mild HTGP patients under negative-ion and positive-ion modes. **(A-1,A-2)** Bubble plots illustrating significantly enriched metabolic pathways identified from differential lipid metabolites in negative-ion (NEG) and positive-ion (POS) modes, respectively. Bubble size represents the number of enriched metabolites within each pathway, whereas color intensity indicates enrichment significance [−log10 (*p*-value)]. **(B-1,B-2)** Pathway–metabolite interaction networks derived from KEGG enrichment analysis in NEG and POS modes, respectively. Nodes represent enriched pathways or differential lipid metabolites, and edges indicate pathway–metabolite associations. Network analysis revealed close interactions among lipid metabolism, inflammatory signaling, necroptosis, and autophagy-related pathways associated with severe disease progression. **(C-1,C-2)** KEGG classification bar charts showing the distribution characteristics of major metabolite subclasses identified in NEG and POS modes, respectively. Lipid metabolites associated with severe progression were predominantly enriched in pathways related to glycerophospholipid metabolism, sphingolipid metabolism, cellular processes, and signal transduction. KEGG, Kyoto Encyclopedia of Genes and Genomes; NEG, negative-ion mode; POS, positive-ion mode; HTGP, hypertriglyceridemic pancreatitis.

In the N_neg mode (S vs. N_neg), thirteen significantly enriched pathways were identified (Figure 11A-1). Among them, retrograde endocannabinoid signaling exhibited the highest enrichment ratio (Ratio = 0.0095), whereas metabolic pathways involved the largest number of enriched metabolites. Additional significantly enriched pathways included glycerophospholipid metabolism, arachidonic acid metabolism, linoleic acid metabolism, alpha-linolenic acid metabolism, pathogenic *Escherichia coli* infection, Kaposi sarcoma-associated herpesvirus infection, GPI-anchor biosynthesis, folate transport and metabolism, choline metabolism in cancer, autophagy-animal, and autophagy-other.

Pathway–metabolite interaction network analysis suggested that glycerophospholipid metabolism occupied a central hub position with extensive interconnectivity with retrograde endocannabinoid signaling and several other enriched pathways (Figure 11B-1), potentially implicating lipid-mediated signaling networks in the biological context of severe HTGP. KEGG classification analysis further revealed that the enriched pathways were predominantly associated with metabolism-related processes, including lipid metabolism (16.67%) and global/overview metabolic maps (16.67%), as well as organismal systems, particularly the nervous system (16.67%) and digestive system (8.33%) (Figure 11C-1).

In the N_pos mode (S vs. N_pos), four significantly enriched pathways were identified (Figure 11A-2), including metabolic pathways, sphingolipid signaling pathway, sphingolipid metabolism, and necroptosis. Notably, sphingolipid metabolism and necroptosis demonstrated the highest enrichment ratios (Ratio ≈ 0.017–0.018) and greater enrichment significance compared with those observed in the N_neg mode (−log10P ≈ 1.0).

Pathway–metabolite interaction network analysis in the N_pos revealed dense clustering among sphingolipid-related metabolites and pathways associated with programmed cell death (Figure 11B-2), suggesting potential involvement of sphingolipid-regulated cell death signaling in severe HTGP, though this requires further experimental validation. KEGG classification analysis further showed that lipid metabolism (23.08%) and global/overview metabolic pathways (23.08%) were the dominant functional categories, followed by signal transduction (15.38%) and cell growth and death (15.38%) (Figure 11C-2).

The two ion-detection modes provided complementary observational perspectives on lipid metabolic alterations associated with disease severity in HTGP. The N_neg mode findings were enriched in pathways related to glycerophospholipid and polyunsaturated fatty acid metabolism, suggesting possible involvement of membrane lipid remodeling and lipid-associated signaling in severe HTGP. The N_pos mode findings pointed to potential dysregulation of sphingolipid-associated programmed cell death pathways. Taken together, these hypothesis-generating observations identify lipid metabolic pathways that may be biologically relevant to severe HTGP and offer pathway-level context supporting the biological plausibility of the clinical predictors identified by the machine learning model. However, given the small sample size (*n* = 15 pairs) and the exploratory nature of this sub-study, these findings should be interpreted with caution. Causal relationships and specific regulatory mechanisms cannot be inferred from KEGG enrichment analysis alone, and experimental validation in larger cohorts or dedicated functional studies will be required to substantiate these pathway-level observations.

## 4 Discussion

In the present study, we developed an explainable machine learning framework integrating routine clinical indicators with exploratory lipidomic pathway evidence for early severity stratification of HTGP at hospital admission. It should be noted that this study employed a cross-sectional design: patients were classified according to the revised Atlanta Classification based on their clinical course during the index hospitalization, and a proportion of patients in the MSAP/SAP group had already met the relevant diagnostic criteria at or shortly after admission. The clinical objective of the model is therefore rapid discrimination of MSAP/SAP from MAP within 24 h of presentation. Using ensemble feature selection strategies, ten robust predictors were identified: IL-6, D-dimer, PCT, ALB, TP, CRP, pleural effusion (PLE), pancreatitis-associated ascitic fluid (PAAF), SIRS, and age. Among all candidate algorithms, the Naive Bayes model achieved the most balanced discriminative performance, with superior sensitivity compared with conventional clinical scoring systems. The Naive Bayes classifier is computationally efficient, robust to relatively small clinical datasets, and requires minimal hyperparameter optimization, supporting practical applicability in emergency triage settings (22). SHAP analysis further enhanced model interpretability by quantitatively characterizing the directional contribution of each predictor.

Different grouping strategies were adopted for the machine learning and lipidomic analyses because these two components addressed distinct objectives. The machine learning model classified patients into MAP versus MSAP/SAP to support severity stratification at admission. In contrast, the lipidomic sub-study — conducted as an exploratory analysis in 15 matched pairs — compared SAP with non-severe patients (MAP/MSAP) using an extreme-phenotype strategy to maximize biological contrast. The lipidomic findings are intended to provide pathway-level biological context for the clinical predictors identified by the machine learning model, and should not be interpreted as independent validation of the ML classification boundary.

Among the identified predictors, inflammatory markers collectively constituted the most prominent cluster. IL-6 exhibited the highest SHAP importance, consistent with its established role in orchestrating the cytokine cascade in acute pancreatitis (23). Excessive free fatty acid (FFA)-mediated lipotoxicity is widely recognized as a core initiating mechanism in HTGP, leading to acinar cell injury and subsequent release of pro-inflammatory cytokines (24–26). Elevated IL-6 further promotes hepatic CRP synthesis and systemic inflammatory propagation, driving persistent SIRS (27). CRP levels exceeding 150 mg/L within 72 h are strongly associated with pancreatic necrosis (28, 29), and the optimal CRP cutoff identified in our study (193.303 mg/L) was largely consistent with previous reports. Persistently elevated PCT levels are considered important indicators of systemic inflammatory escalation and necrotizing pancreatitis progression (30, 31). In the exploratory lipidomic sub-study, pathway enrichment analysis identified alterations in arachidonic acid, linoleic acid, and alpha-linolenic acid metabolism, consistent with polyunsaturated fatty acid dysregulation previously implicated in inflammatory amplification (32–34). These observations provide hypothesis-generating biological context supporting the plausibility of the inflammatory predictors, though causal inference requires experimental validation.

D-dimer was identified as one of the most influential predictors, reflecting early activation of coagulation and fibrinolytic imbalance (35). Severe inflammatory responses are known to induce endothelial injury and microvascular thrombosis, whereas hypertriglyceridemia-associated metabolic disturbances further impair fibrinolysis, aggravating pancreatic ischemia (36, 37). In the lipidomic sub-study, glycerophospholipid metabolism was among the enriched pathways. Published evidence suggests that phospholipase A2-mediated phospholipid degradation can generate products capable of disrupting endothelial integrity (38, 39); whether this pathway contributes to coagulation activation in the present cohort remains a hypothesis that warrants future experimental investigation.

ALB and TP were both negatively associated with severe disease, reflecting the extent of vascular permeability and plasma protein extravasation (40, 41)^.^ Pleural effusion and PAAF were retained as independent predictors in the final model. We acknowledge that these findings may be present at or shortly after admission in a subset of patients, given the cross-sectional nature of this study; their inclusion reflects their established value as markers of systemic disease burden in HTGP rather than as antecedent risk factors. SIRS represents a pivotal indicator of systemic inflammatory escalation and is closely associated with adverse clinical outcomes (42, 43).

In the exploratory lipidomic sub-study, positive-ion mode enrichment additionally identified alterations in sphingolipid metabolism and necroptosis-related pathways, while retrograde endocannabinoid signaling demonstrated the highest enrichment ratio in negative-ion mode. These observations are consistent with prior literature implicating ceramide-mediated cell death and endocannabinoid signaling in inflammatory conditions (44–46), and may suggest candidate biological mechanisms in severe HTGP. However, given the small sample size (*n* = 15 pairs) and exploratory design of this sub-study, these findings are strictly hypothesis-generating and cannot be interpreted as mechanistic evidence without dedicated experimental validation.

Enrichment of autophagy-related pathways in the lipidomic analysis is consistent with published evidence that impaired autophagic flux contributes to intracellular trypsinogen activation and acinar cell injury in acute pancreatitis (47, 48). This observation may offer a potential biological context for the predictive contribution of PCT, though mechanistic confirmation remains to be established.

Younger age was associated with higher disease severity, with an optimal cutoff of 38.858 years. This is consistent with epidemiological data demonstrating that HTGP disproportionately affects younger individuals with obesity, metabolic syndrome, and related lifestyle risk factors (49–51), who may exhibit more pronounced lipotoxic and inflammatory responses.

Several aspects distinguish the present study from previous investigations in HTGP. First, the model was designed for rapid severity stratification within 24 h of admission using routinely available clinical indicators, addressing a practical triage need. Second, the integration of exploratory lipidomic pathway evidence provides biological context for the machine learning-derived predictors, improving interpretability and illustrating how explainable artificial intelligence may serve not only as a discriminative tool but also as a framework for generating mechanistic hypotheses in metabolically driven inflammatory diseases. Nonetheless, these strengths should be considered in light of the study’s limitations, particularly the absence of external validation.

Several limitations should be acknowledged. First, and most importantly, this was a single-center retrospective study, and the model has not been externally validated in an independent cohort. The performance metrics reported here were derived from a single-center internal test set and should be regarded as preliminary; generalizability and robustness cannot be established without confirmation in independent, multicenter cohorts with varying patient demographics and clinical practice patterns. Second, the cross-sectional severity stratification design means that a proportion of patients in the MSAP/SAP group may have met diagnostic criteria at or near the time of admission; the model should therefore be interpreted as a severity identification tool rather than a progression predictor. Third, the relatively limited sample size may have introduced selection bias and could affect predictor stability, further underscoring the need for external validation. Fourth, the lipidomic sub-study was based on only 15 matched pairs; all pathway-level findings should be regarded as hypothesis-generating, as KEGG enrichment analysis cannot establish causal relationships or confirm specific regulatory mechanisms. Fifth, the grouping strategies used in the machine learning and lipidomic analyses were not identical, and the lipidomic findings should not be interpreted as direct validation of the ML classification boundary. Furthermore, although the Naive Bayes model demonstrated favorable discrimination, its calibration slope (0.2009) indicated compression of predicted probabilities, which may limit the accuracy of individual absolute risk estimation. Recalibration using Platt scaling or isotonic regression in a larger external cohort is recommended to improve probabilistic reliability.

In conclusion, we developed an explainable Naive Bayes framework integrating routine clinical indicators with exploratory lipidomic pathway evidence for early severity stratification of HTGP at hospital admission. The identified predictors converged on interconnected clinical axes including inflammatory amplification, coagulation dysfunction, and endothelial leakage. The lipidomic sub-study provided complementary, hypothesis-generating biological context supporting the plausibility of these predictors, while identifying sphingolipid metabolism, programmed cell death pathways, and endocannabinoid signaling as candidate mechanisms warranting future experimental investigation. These findings represent preliminary evidence from a single-center retrospective cohort; given the absence of external validation, the present framework should be regarded as proof-of-concept, and its performance and generalizability remain to be confirmed in independent multicenter cohorts. If validated, this integrative approach may offer a practical paradigm for biologically grounded, explainable risk stratification in metabolically driven inflammatory diseases.

## Data Availability

The raw data supporting the conclusions of this article will be made available by the authors, without undue reservation.
